# Supplementary material for: Tribological Properties of Nitrate Graphite Foils
Source: Nanomaterials (Basel). 2024 Sep 15;14(18):1499. doi: 10.3390/nano14181499 (PMC11434555; doi:10.3390/nano14181499)
Supplement: Supplementary file 1 [file nanomaterials-14-01499-s001.zip › X-ray data/macrostress/0001/over500/dens_16/TD/213.doc]

**Analysis Results**

**General Information**

| Analysis date | 2024/05/17 09:16:53 | | |
| --- | --- | --- | --- |
| Sample name | 0001_500_16_RD | Measurement date | 2024/05/16 13:59:05 |
| File name | 213.raw | Operator | Administrator |
| Comment | Psi = 0.000 | | |

**Measurement profile**

**Measurement conditions**

| X-Ray | 40 kV , 30 mA | Scan speed / Duration time | 1.0000 sec |
| --- | --- | --- | --- |
| Goniometer |  | Step width | 0.0200 |
| Attachment | - | Scan axis | 2theta/theta |
| Filter | K-beta filter | Scan range | 84.0000 - 90.0000 |
| CBO selection slit | BB | Incident slit | 2/3?‹\|2/3deg. |
| Diffrected beam mono. |  | Length limiting slit | - |
| Detector | Scintillation counter | Receiving slit #1 | Open |
| Scan mode | STEP | Receiving slit #2 | 0.30mm |

**Qualitative analysis results**

| Phase name | Formula | Figure of merit | Phase reg. detail | DB card number |
| --- | --- | --- | --- | --- |

| Phase name | Formula | Space group | Phase reg. detail | DB card number |
| --- | --- | --- | --- | --- |

**Peak list**

| No. | 2-theta(deg) | d(ang.) | Height(cps) | FWHM(deg) | Int. I(cps deg) | Size(ang.) | Phase name |
| --- | --- | --- | --- | --- | --- | --- | --- |
| 1 | 86.923(8) | 1.11981(8) | 2839(53) | 0.621(9) | 2777(28) | 180(2) | Unknown |
| 2 | 86.924(9) | 1.11980(9) | 2577(51) | 0.645(9) | 2615(30) | 173(3) | Unknown |
| 3 | 86.914(12) | 1.11991(12) | 1853(43) | 0.713(11) | 1992(35) | 157(2) | Unknown |
| 4 | 86.917(16) | 1.11987(16) | 1125(34) | 0.830(13) | 1353(41) | 135(2) | Unknown |
| 5 | 86.85(3) | 1.1206(3) | 612(25) | 0.97(2) | 827(25) | 115(3) | Unknown |
| 6 | 86.91(4) | 1.1200(4) | 291(17) | 1.12(3) | 400(28) | 100(3) | Unknown |

**Parameters used for WPPF**

Profile parameters

| Common parameter | Background | Data | 213-1 |
| --- | --- | --- | --- |
|  |  | Function name | B-spline |
|  |  | param0 | 895.61892262545337 |
|  |  | param1 | 953.95320053988939 |
|  |  | param2 | 884.92824989313488 |
|  |  | node0 | 84 |
|  |  | node1 | 90 |
| Common parameter | Peak shift |  |  |
|  |  | Function name | Shift axial displacement |
|  |  | param0 | 0 |
|  |  | param1 | 0 |
|  |  | param2 | 0 |

Structure parameters

| Data set name | Phase Name | Element | x | y | z | Occupancy | Temperature factor |
| --- | --- | --- | --- | --- | --- | --- | --- |

| Data set name | Rwp | Rp | Re | S | Chi^2 | Maximum shift/e.s.d. |
| --- | --- | --- | --- | --- | --- | --- |
| 213-1 | 0 | 0 | 0 | 0 | 0 | 0 |
| 213-2 | 0 | 0 | 0 | 0 | 0 | 0 |
| 213-3 | 0 | 0 | 0 | 0 | 0 | 0 |
| 213-4 | 0 | 0 | 0 | 0 | 0 | 0 |
| 213-5 | 0 | 0 | 0 | 0 | 0 | 0 |
| 213-6 | 0 | 0 | 0 | 0 | 0 | 0 |
| 213-7 | 0 | 0 | 0 | 0 | 0 | 0 |

**Lattice constants**

Angular correction

Analysis results

| Data set name | a(A) | b(A) | c(A) | alpha(deg) | beta(deg) | gamma(deg) |
| --- | --- | --- | --- | --- | --- | --- |

| Phase name | a(A) | b(A) | c(A) | alpha(deg) | beta(deg) | gamma(deg) | V(A^3) |
| --- | --- | --- | --- | --- | --- | --- | --- |

LCGraph.emf

**Crystallinity**

| Data set name | Crystallinity(%) |
| --- | --- |

CrystallinityGraph.emf

**Crystallite size and lattice strain**

Williamson-Hall method

| Data set name | Crystallite size(A) | Strain(%) |
| --- | --- | --- |

| Phase name | Crystallite size(A) | Distribution RSD | Strain(%) | Distribution type |
| --- | --- | --- | --- | --- |

CSSGraph.emf

**Quantitative analysis results (RIR)**

RIRGraph.emf

**Quantitative analysis results (WPPF)**

**Quantitative analysis results (standard addition method)**

Calibration data

QuantityCalibration.emf

**Quantitative analysis results (External Standard method)**

Calibration data

QuantityCalibration.emf

**Quantitative analysis results (internal standard method)**

Calibration Data

QuantityCalibration.emf

**Stress**

Stress constants

| Phase name | Unknown | Young's modulus(MPa) | 184500 |
| --- | --- | --- | --- |
| Stress-free 2-theta angle(deg) | 87.010 | Poisson's ratio | 0.300 |
| Wavelength(ang) | CuKa (1.541836) | Stress constant(MPa/deg) | -1304.89200 |

Analytical conditions

|  | Psi angle(deg) | (sin(psi))^2 | 2-theta(deg) |
| --- | --- | --- | --- |
| * | 0 | 0 | 86.923(8) |
| * | 5 | 0.008 | 86.924(9) |
| * | 10 | 0.03 | 86.914(12) |
|  | 15 | 0.067 | 86.917(16) |
| * | 20 | 0.117 | 86.85(3) |
|  | 25 | 0.179 | 86.91(4) |

Analysis results

|  | Stress +/-Reliability(MPa) | Fomula | 2-theta(Psi=0)(deg) |
| --- | --- | --- | --- |
| + | 806 +/- 149 | Y=-0.62X+86.926 | 86.923(8) |
| - | - | Y=-0.62X+86.926 |  |

**Cluster analysis results**

Dendrogram

ClusterDendrogram.emf

Measurement profiles

Cluster

Sample well

ClusterSamplePlate.emf

PCA view

ClusterPCA3DGraph.emf

Eigenvalue graph

ClusterPCAGraph.emf

**Crystallite size distribution analysis results**

Crystallite size distribution

Crystallite size distribution graph

**Crystal structure analysis results**

Indexing

| Phase name | Formula | Figure of merit | Phase reg. detail | DB card number |
| --- | --- | --- | --- | --- |

Quantitative analysis results

Lattice information

| Phase name | a(A) | b(A) | c(A) | alpha(deg) | beta(deg) | gamma(deg) | V(A^3) |
| --- | --- | --- | --- | --- | --- | --- | --- |

| Phase name | Space group | Z | Z' | Calc. density(g/cm^3) |
| --- | --- | --- | --- | --- |

Structure determination

Refinement

Measurement range: 84.0000-90.0000deg Refinement range: 84.0000-90.0000deg (1.09 A)

Number of refined parameters: 0

| Phase name | Atomic coords | # of indep. reflns |
| --- | --- | --- |

Rwp = -	S = -

Crystal structure

CrystalGraph.emf
